# Effect of educational intervention based on health belief model on accident prevention behaviours in mothers of children under 5-years

**DOI:** 10.1186/s12905-021-01573-1

**Published:** 2021-12-27

**Authors:** Elnaz Moridi, Zahra Fazelniya, Asiyeh Yari, Tahereh Gholami, Pooyan Afzali Hasirini, Ali Khani Jeihooni

**Affiliations:** 1 Department of Public Health, School of Health, Fasa University of Medical Sciences, Fasa, Iran; 2 Department of Nursing, Yasuj Branch, Islamic Azad University, Yasuj, Iran; 3 Department of Health Education and Health Promotion, School of Health, Hormozgan University of Medical Sciences, Bandar Abbas, Iran; 4 Department of Public Health, School of Health, Kermanshah University of Medical Sciences, Kermanshah, Iran; 5 Nutrition Research Center, Department of Public Health, School of Health, Shiraz University of Medical Sciences, Shiraz, Iran

**Keywords:** Health belief model, Accidents, Mothers

## Abstract

**Background:**

As the public health problems, accidents are the most important causes of child mortality. The present study aimed to determine the effect of educational intervention based on health belief model on accident prevention behaviors in mothers of children under 5-years of age.

**Methods:**

This quasi-experimental study was conducted on 200 mothers in Fasa city who were purposefully selected and randomly divided into two groups of intervention and control. Data collection tools were demographic characteristics and health belief model questionnaire. Questionnaires were completed twice before and 3 months after the intervention. After the pre-test, the educational intervention was performed through 6 sessions of 30–35 min in a WhatsApp group. Data were analyzed using SPSS 22 through Chi-square test, independent *t*-test and paired *t*-test (*p* = 0.05).

**Results:**

The mean age of mothers in the experimental and control groups was 30.14 ± 4.35 and 31.08 ± 4.31 years. Mean score of awareness, perceived sensitivity, perceived severity, perceived benefits, perceived self-efficacy, cues to action, and accident prevention behaviors significantly increased 3 months after the intervention.

**Conclusion:**

This study showed the effectiveness of educational intervention based on health belief model on accident prevention behaviors in mothers of children under 5-years of age.

## Background

Accidents in children are among the important public health problems [1]. Studies also showed that the first leading cause of death in the first five years of life is accidents, more than 95% of which occur in low- and middle-income countries [2]. According to The United States Centers for Disease Control and Prevention, unintentional accidents are the fifth leading cause of death in children under 1-year of age and the first leading cause of death in children aged 1–4 [3]. In India, 10–15% of deaths, 20–30% of hospitalizations, and 20% of disabilities in children are due to accidents [4]. In Japan, accidents have been the leading cause of death in children for the past 50 years [5]. According to Iran Ministry of Health and Medical Education, 19–37% of child deaths are due to unintentional and often preventable accidents such as fall, burning, road accidents, drowning, and poisoning [6]. Treating the injuries of accidents and lifelong costs caused by the complications for children have a significant economic burden for the health system of countries [7, 8]. If the scope of attention to the health consequences of accidents becomes wider, most of the consequences would not be limited to immediate injuries caused by the accident. Any accident leading to physical injury in most cases leads to physical illness, psychological complications, and social damage [9]. Given the importance of the Millennium Development Goals to reduce the mortality rate of children under 5-years of age and considering the fact that the survival of children is a global scale to measure the rate of development and well-being, investment in child health is not only a matter of human rights but also a necessary economic decision [10, 11]. According to studies, 90% of injuries caused by accidents in children are predictable and educating mothers as a key person in child care has a great impact on the prevention of such injuries [12, 13]. This is possible to some extent through the use of educational models. Choosing the right model causes the program to start in the right direction [14].

The health belief model (HBM) tries to explain and predict health behaviors [15]. The model emphasizes how people perceive motivation to create new behaviors. It generally emphasizes the attitude of individuals and considers a change in attitude leading to a change in behavior [16]. The HBM comprises of six constructs: perceived sensitivity, perceived benefits, perceived barriers, perceived severity, cues to action, and self-efficacy. Based on the main hypotheses of the model, the person adopts his/her health behavior based on the following factors: increased susceptibility to a disease or harmful condition as a result of engaging in a particular behavior, increasing one’s perception of the severity of the injury as a result of a particular illness or behavior, increasing one’s perception of benefits of health behavior, reducing barriers and costs as a result of health behavior, having the necessary ability to follow health behavior, and accelerating the adoption of health behavior [17–21]. The results of a study by Heidarikia et al. showed that educational programs based on the HBM have a significant effect on improving beliefs and safe behaviors preventing children from accidents by mothers [22]. In a study by Meimantabadi et al., education based on the HBM had a positive effect on improving safety knowledge, changing attitudes, and improving mothers' performance regarding injuries of children under 5 years of age [23]. In a study by Fathi et al., the mean score of HBM constructs were reported above-average. In this study, there was a negative significant correlation between self-efficacy and perceived barriers and a negative significant correlation between perceived barriers and performance [12]. Educating mothers could be helpful in reducing mortality and possible complications from accidents in children; therefore, the present study aimed to investigate the effect of educational intervention based on the HBM on accident preventing behaviors in mothers of children under-5 years of age.

## Methods

### Participants

This quasi-experimental study was conducted on 200 mothers of children under 5-years of age who referred to health centers of Fasa city, Iran, 2020–2021.

### Sampling

**Fig. 1:**
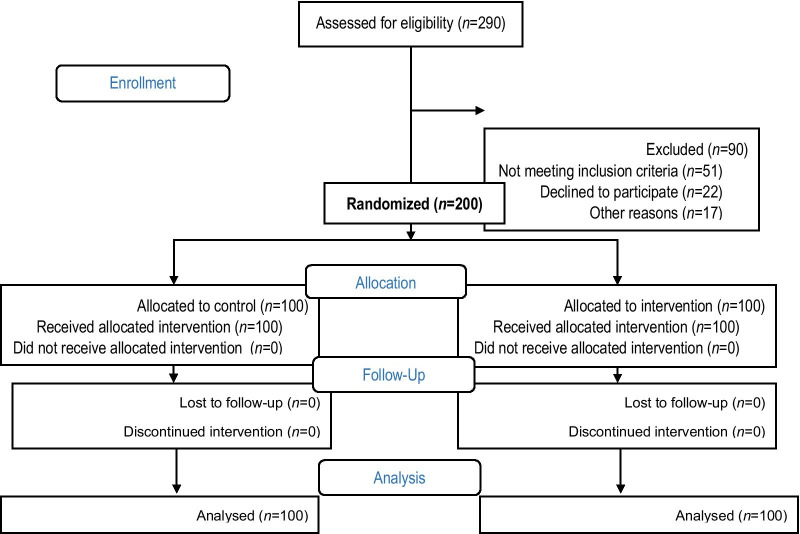
Flow chart of the study

Out of 6 urban health centers, 2 were randomly selected (1 for the experimental group and 1 for the control group). Sampling was performed randomly and according to the family number of the women's health file under the auspices of the centers, and then with the coordination of the health center officials, a WhatsApp group was formed. While getting to know the subjects and stating the goals of the study, informed consent was obtained from the participants. The sample size of the study was considered to be 200 according to the same study [12], with a standard deviation of 18.8, reliability level of 95%, and 80% power. Figure 1 presents the study flow chart.

Inclusion criteria were mothers of children under 5-years of age, willingness to participate in the study, able to read and write. Exclusion criteria were absence from more than one training session, unwillingness to participate in the study, failure to complete the questionnaire, and home safety checklist.

In this study, the data was collected self-reportedly through a standard questionnaire taken from the study of Poorolajal et al. [24] and Dehghani et al. [25], including demographic characteristics (age of mothers, age of children, family dimension, mother's occupation, father's occupation, monthly household income, mother's education, father's education, and children's gender) and accident prevention questionnaire based on the HBM, comprises of 15 questions on awareness, 5 questions on perceived barriers, 5 questions on perceived benefits, 5 questions on perceived sensitivity, 5 questions on perceived severity, 4 questions on self-efficacy, 4 questions on cues to action, and 20 questions on performance.

Perceived sensitivity, severity, benefits, and barriers; cues to action and self-efficacy were scored between 1 and 6 (ranging from completely disagree (1), disagree (2), rather disagree (3), rather agree (4), agree (5), to completely agree (6)).

Here are some examples of commonly used questions. Awareness: Which case is one of the most severe and common injuries in children under five? a) Scratching b) Bone fractures and joint dislocations C) cuts and wounds d) trauma. Perceived Susceptibility: My child may be moderately injured. (Moderate injury: requires less than 24 h of outpatient treatment in medical centers: Strongly agree/Agree/Rather agree/Rather disagree/Disagree/Strongly disagree. Perceived severity: Moderate injuries cause temporary disability in my child's activities. Perceived Benefits: Monitoring my child's activities inside the house helps to maintain his health. Perceived Barriers: I get tired of constantly monitoring my child's activities. Cues to action: Health centers advise me to follow the principles of safety and prevention of child injury. Self-Efficacy: I am confident that I can monitor my child's activities at all times. Performance: The surfaces of the house are smooth and clean and not slippery: Yes/No/ I do not know.

The reliability of the questionnaire was measured as follows: sensitivity (0.72), severity (0.71), benefits (0.81), barriers (0.63), self-efficacy (0.77), and cues to action (0.73). Also, the questionnaire had an Alpha Cronbach of 0.84. The purpose of the study and how to do the work were explained to participants and the staff of the health centers. The questionnaire was completed by two experimental and control groups.

Based on the results of the pre-test and the study of Dehghani et al. [25], an initial needs assessment was performed, in which most of the cases of mothers' poor performance in preventive behaviors inside and outside the home were examined. The educational content was prepared based on the HBM. The predictive structures of this study were the perceived barriers, perceived susceptibility, perceived benefits, and self-efficacy. The educational intervention for the experimental group consisted of 6 virtual training sessions of 30–35 min in the form of lectures, questions and answers, group discussions, using educational images, video clips, and PowerPoint. The training program was conducted by a doctor of health education and health promotion and a medical student in collaboration with two non-communicable disease experts. The details of the training sessions are presented in Table 1.

**Table 1:** The details of the training sessions

Sessions	Educational content title
First session	Reports were virtually presented on children's growth and development
	Injuries caused by accidents and their impact on the child's development
Second session	Real accidents in the world and Iran
	The seriousness of complications and injuries caused by accidents
Third session	A group discussion was also held to overcome the barriers to safe behaviours
	Each mother in the intervention group was asked to describe the history of accidental injury in their child that they have experienced in the past
Fourth session	To explain how the injury and their actions to prevent its recurrence
	The purpose of this work was to raise the concerns of mothers and further emphasized the benefits and importance of safety measures
Fifth session	Risk factors, general acciden* t*-causing conditions and safe behaviors in preventing possible accidents outside the home were explained to mothers and for home safety
	Necessary trainings were provided to prevent falling from stairs and windows, collecting sharp tools at home
Sixth session	Using personal protective equipment by children during play
	To maintain and improve the activity of the experimental group, they received an instructional message per week

Two follow-up Phone tracking were held for mothers one month and 3 months after the educational intervention. Three months after the educational intervention, both experimental and control groups completed the questionnaire. For the control group, at the end of the study, the entire educational content was sent as a virtual educational package. For ethical considerations, while obtaining permission from the ethics committee of Fasa University of Medical Sciences (design code: 97488 - IR.FUMS.REC.1399.171 ethics code), a written consent was obtained from participants and the goals, importance and necessity of the study was explained to them. Participants were also assured that their information would remain confidential. Data were analysed using SPSS 22 using Chi-square, independent *t*-test and paired *t*-test and the significance level was considered 0.05.

## Results

In this study, 200 mothers of children under 5-years of age participated. The mean age of mothers in the experimental and control groups was 30.14 ± 4.35 and 31.08 ± 4.31 years, respectively (*p* = 0.212). The mean age of children in the experimental and control groups was 36.12 ± 50.13 and 35.13 ± 96.02 months, respectively (*p* = 0.218). The mean the number of family members in the experimental and control groups was 3.92 ± 1.04 and 3.87 ± 1.14, respectively (*p* = 0.209). Chi-square test showed no significant difference between the two experimental and control groups in terms of mother’s occupation, father’s occupation, monthly household income, mother’s education, father’s education and children’s gender (Table 2).

**Table 2:** Comparison of frequency distribution of demographic variables between experimental and control groups

Variables	Experimental group		Control group		*p* value
	Number	Percentge	Number	Percentage	
Mother’s occupation					
Housewife	81	81	84	84	0.197
Employed	19	19	16	16	
Father’s occupation					
Employed	22	22	27	27	0.186
Worker	12	12	14	14	
Self-employed	40	40	37	37	
Other	26	26	22	22	
Monthly household income					
< 30 million Rials	40	40	37	37	0.180
30–60 million Rials	35	35	34	34	
˃60 million Rials	25	25	29	29	
Mother’s education					
Primary school	10	10	13	13	0.192
Secondary school	21	21	20	20	
High school	48	48	44	44	
College	21	21	23	23	
Father’s education					
Primary school	8	8	5	5	0.102
Secondary school	18	18	20	20	
High school	42	42	50	50	
College	32	32	25	25	
Children’s gender					
Male	53	53	49	49	0.106
Female	47	47	51	51	

The results showed that before the educational intervention, there was no significant difference between the experimental and control groups in terms of awareness, perceived sensitivity, perceived severity, perceived benefits, perceived barriers, self-efficacy, cues to action and performance. However, after the educational intervention, the experimental group showed a significant increase in all the variables except perceived barriers (Table 3).

**Table 3:** Comparison of mean scores of awareness, perceived sensitivity, perceived severity, perceived benefits, perceived barriers, self-efficacy, cues to action and performance in experimental and control groups before and 3 months after educational intervention

Variable	Group	Before the intervention	6 months after the intervention	*p*-value
Awareness	Experimental	44.15 ± 8.23	77.12 ± 8.97	0.001
	Control	46.22 ± 8.10	47.39 ± 38.14	0.282
	*p*-value	0.253	0.001	
Perceived sensitivity	Experimental	28.16 ± 5.14	70.28 ± 8.33	0.001
	Control	27.59 ± 4.97	29.10 ± 4.22	0.259
	*p*-value	0.277	0.001	
Perceived severity	Experimental	38.25 ± 6.72	78.28 ± 7.49	0.001
	Control	36.70 ± 6.92	39.04 ± 6.61	0.273
	*p*-value	0.217	0.001	
Perceived benefits	Experimental	41.16 ± 7.42	80.10 ± 8.25	0.001
	Control	43.11 ± 7.30	45.23 ± 7.32	0.216
	*p*-value	0.220	0.001	
Perceived barriers	Experimental	68.86 ± 8.23	22.49 ± 4.44	0.001
	Control	65.88 ± 8.34	64.09 ± 8.27	0.287
	*p*-value	0.202	0.001	
Perceived self-efficacy	Experimental	29.82 ± 5.33	71.86 ± 8.26	0.001
	Control	30.82 ± 5.91	32.14 ± 5.83	0.284
	*p*-value	0.295	0.001	
Cues to action	Experimental	34.12 ± 4.25	70.18 ± 8.29	0.001
	Control	33.39 ± 4.38	35.92 ± 4.46	0.199
	*p*-value	0.293	0.001	
Performance	Experimental	32.28 ± 4.55	69.94 ± 8.08	0.001
	Control	33.80 ± 4.60	35.12 ± 4.65	0.257
	*p*-value	0.288	0.001	

## Discussion

Since prevention of home accidents in children under the age of five is an important issue and mothers have an effective role in this regard, the present study aimed to determine the effect of educational intervention based on health belief model on accident prevention behaviors in mothers of children under 5-years of age in Fasa city. The results of the present study showed that the mean score of awareness in the intervention group increased significantly compared to the control group; therefore, educational content and increasing information on children's growth and injuries caused by accidents and their impact on child development increased mothers' awareness on accident preventing behaviors. This is consistent with the results of a study by Fathi et al. [12]. In a study by Razi et al. [26], the necessity of educating mothers on danger signs in children was emphasized. In a study by Alavi et al. [27], effect of increasing awareness on avoiding high-risk situations was investigated. Sreeamareddy et al. [28] also reported that mothers' awareness on danger signs in children is insufficient and that their ability to diagnose and care for danger signs needs to be improved. However, it should be noted that increasing awareness alone can’t change performance. Therefore, in order to change performance, besides awareness, interventions should aim to improve people's attitudes [29]. Mean score of perceived threat (Perceived Sensitivity and severity) was not significantly different between the intervention and control groups before the educational intervention, however, after the educational intervention, the score increased. Therefore, educational intervention was found to be effective in increasing the construct. Expressing the consequences of accidents in children, including amputations, mental problems, and financial burden, improved mothers' understanding; therefore, recounting the story of accidents in children by mothers, providing statistics of casualties and complications, reporting real accidents, and watching documentaries of real experiences were effective in increasing the perceived severity and sensitivity. In similar studies, lectures, group discussions, and child death scenarios increased the perceived threat in mothers [12, 30, 31]. Also, the results of the present study showed that the perceived benefits were significantly different between the two groups after the educational intervention. The increase in perceived benefits is consistent with the results of studies by Asadpour et al. [32]. Shojaeizadeh et al. [33] and Pirzadeh et al. [34]. The reason could be due to the effectiveness of educational intervention using methods such as group discussion, question and answer, and providing suggested solutions to reduce risk and make mothers feel more responsible. Therefore, mothers' trust in the benefits of the proposed methods to reduce the deterioration of accidents, paying attention to the recommendations provided regarding monitoring the child's activities inside and outside the house, paying attention to the safety of the home environment and the equipment with which the child deals, and using strategies in the event of accidents to reduce complications led to understand the benefits of accident preventing behaviours. In the present study, the mean score of perceived barriers decreased in the intervention group. These results were consistent with the results of various studies [30, 32, 33, 35]. Since mothers faced barriers in adopting preventive behaviors such as time-consuming constant supervision of the child, costly and time-consuming installation of doors and windows, and repairing stairs, it was tried to provide the necessary training to understand the benefits of preventive behaviors. In order to reduce the barriers, different methods were used including sending voice messages, group discussion, brainstorming, recounting the past events and measures taken to prevent recurrence of accidents.

The results of the present study showed that the mean score of the cues to action in the intervention group increased significantly after the intervention. The reason was the effectiveness of the educational content provided to mothers. In studies by Fathi et al. [12] and Sajjadi et al. [36] cues to action, including physicians, staff of health centers, relatives, friends, spouse and mass media, were found to be effective in increasing the preventive behaviors. These cues were similar to the results of the present study; therefore, sending various messages as reminders, interpersonal interactions between health education specialists and health workers and mothers, and strong media communication in the present study as cues had a great impact on increasing the mean score of the cues to action. The results of the present study showed that the mean score of self-efficacy was higher than the average and increased significantly in the intervention group. The reason could be due to the effectiveness of educational intervention in increasing the self-efficacy of mothers under educational intervention. In the present study, one of the objectives was to increase performance. The higher the mother's performance, the better their preventive behaviors. The mean score of performance in the intervention group increased significantly, indicating the effectiveness of the educational intervention. The results of many studies are consistent with the results of the present study [33–36].

## Conclusion

The results of the present study showed the positive effect of an educational program based on the health belief model on improving perceived sensitivity, perceived severity, perceived benefits, perceived barriers and self-efficacy of intervention group, followed by an increase in accident preventing behaviors. Considering the effective role of mothers, it seems that designing educational interventions based on the HBM and training through mass media can play a decisive role in reducing home injuries in children.

## Limitations

The study was performed during the Covid-19 epidemic; therefore, the educational intervention was held virtually. The behavior of the participants was assessed by using a questionnaire and a phone call. The participants cooperated until the end of the study and did not leave the study.

## Data Availability

All supporting data are available through the corresponding author.

## Abbreviation

HBM: Health belief model
